# Catalyst for change: Psilocybin’s antidepressant mechanisms—A systematic review

**DOI:** 10.1177/02698811241312866

**Published:** 2025-01-20

**Authors:** Joshua Liebnau, Felix Betzler, André Kerber

**Affiliations:** 1 Division of Clinical Psychological Intervention, Freie Universität Berlin, Berlin, Germany; 2 Department of Psychiatry and Neurosciences, Charité—Universitätsmedizin Berlin, CCM, Corporate Member of Freie Universität Berlin and Humboldt Universität zu Berlin, Berlin, Germany

**Keywords:** Psilocybin, depression, mechanisms of change, common factors of psychotherapy, functional connectivity, default mode network, experiential avoidance, connectedness

## Abstract

**Background:**

Recent clinical trials suggest promising antidepressant effects of psilocybin, despite methodological challenges. While various studies have investigated distinct mechanisms and proposed theoretical opinions, a comprehensive understanding of psilocybin’s neurobiological and psychological antidepressant mechanisms is lacking.

**Aims:**

Systematically review potential antidepressant neurobiological and psychological mechanisms of psilocybin.

**Methods:**

Search terms were generated based on existing evidence of psilocybin’s effects related to antidepressant mechanisms. Following Preferred Reporting Items for Systematic Reviews and Meta-Analysis guidelines, 15 studies were systematically reviewed, exploring various therapeutic change principles such as brain dynamics, emotion regulation, cognition, self-referential processing, connectedness, and interpersonal functioning.

**Results:**

Within a supportive setting, psilocybin promoted openness, cognitive and neural flexibility, and greater ability and acceptance of emotional experiences. A renewed sense of connectedness to the self, others, and the world emerged as a key experience. Imaging studies consistently found altered brain dynamics, characterized by reduced global and within default mode network connectivity, alongside increased between-network connectivity.

**Conclusions:**

Together, these changes may create a fertile yet vulnerable window for change, emphasizing the importance of a supportive set, setting, and therapeutic guidance. The results suggest that psilocybin, within a supportive context, may induce antidepressant effects by leveraging the interplay between neurobiological mechanisms and common psychotherapeutic factors. This complements the view of purely pharmacological effects, supporting a multileveled approach that reflects various relevant dimensions of therapeutic change, including neurobiological, psychological, and environmental factors.

## Introduction

Depression can be characterized as a disease of disconnection: from the sense of self, significant others, the environment, emotions, and behaviors (Christensen et al., 2022; Osler, 2022; Wickramaratne et al., 2022). Symptoms of depression further maintain disconnection by decreasing individuals’ motivation to engage with novel positive stimuli, favoring negative information (Winer and Salem, 2016). Recent research suggests hyperconnectivity within the default mode network (DMN) as an underlying neurobiological factor of rigid functioning and negativity bias (Chou et al., 2023; Guha et al., 2021; Zhou et al., 2020). Decreased openness for novel stimuli poses a significant challenge for therapeutic change processes, particularly in treating depression. About 30% of those diagnosed with depression fulfill the criteria of treatment-resistant depression (TRD), leading to disproportionately high healthcare costs and significant mental distress, highlighting the great need for novel treatment approaches (Zhdanava et al., 2021).

Classic psychedelics such as psilocybin have been used by humans for thousands of years in indigenous medicine traditions (Lowe et al., 2021). In the 1960s, researchers enthusiastically investigated the use of psychedelics in treating several mental disorders, such as depression, anxiety disorders, and alcohol addiction (Passie et al., 2002). After psilocybin was listed as a Schedule I drug by the United Nations in 1971, all research came to an end despite promising early findings of significant antidepressant effects (Lowe et al., 2021). More than 50 years after the prohibition, research was revived with a series of clinical trials, initiating the so-called *psychedelic renaissance* (Kelly et al., 2022; Pearson et al., 2022).

Psilocybin, a psychedelic prodrug compound, is naturally produced by over 200 mushrooms (Nichols, 2020). Its active metabolite psilocin can cross the blood–brain barrier and exerts an agonistic effect on serotonin 5-HT_2A_ receptors (MacCallum et al., 2022). 5-HT_2A_ receptors are a key component of the serotonergic system, which is associated with a wide range of functions including emotion and mood regulation, complex cognitive processing, perception, and learning (Švob Štrac et al., 2016). 5-HT_2A_ receptors are most densely expressed in the prefrontal cortex, limbic system, basal ganglia, and claustrum (Hensler, 2012; Švob Štrac et al., 2016). Psilocybin induces various subjective effects including changes in self-referential cognition, emotional states, and sensory experiences (Studerus et al., 2011). Collectively, the effects are known to acutely alter individuals’ sense of consciousness, inducing phenomena described as *oceanic boundlessness, ego dissolution*, or *visionary restructuralization*, among others (Studerus et al., 2010). Other subjective effects commonly referred to as *mystical-type experiences* encompass profound feelings of unity, noetic quality, positive mood, transcendence of time and space, and ineffability, among others (MacLean et al., 2012). Recent studies have placed particular emphasis on feelings of unity and connectedness with oneself, others, and the world (Watts et al., 2022).

At the neural level, psilocybin is believed to promote neuroplasticity and change functional connectivity (FC) by reducing within-network FC and increasing between-network FC, including in the DMN (Gattuso et al., 2023; Ly et al., 2018). These changes in FC are linked to alterations in brain modularity, a measure of how distinctly brain regions or networks interact (Sporns and Betzel, 2016). Higher brain modularity reflects more segregated, specialized network activity, which may enhance specific cognitive functions (Chaddock-Heyman et al., 2020). However, excessive modularity can lead to rigid functioning, whereas reduced modularity supports increased network integration and flexibility (Gattuso et al., 2023; Sporns and Betzel, 2016). Together, psilocybin’s impact on brain dynamics may thus foster more flexible and less rigid brain functioning.

The effects vary greatly depending on the individual, dosage, and the psychological, emotional, and environmental context, collectively known as *set and setting* (Hartogsohn, 2017; MacCallum et al., 2022). It is crucial to understand that increased sensitivity to context opens the door for both functional and dysfunctional change, depending on the context’s nature. In a hostile environment, increased context receptivity is likely to promote anxiety and increase the risk of adverse events, whereas in a supportive setting, it may help to overcome rigid depressive states previously limiting treatment efficacy (Gründer et al., 2024b).

A growing body of evidence suggests that psilocybin-assisted therapy exerts its antidepressant effects through mechanisms of change known from psychotherapeutic processes (Greenway et al., 2020; Gründer et al., 2024a; Nayak and Johnson, 2021; Wolff et al., 2024). The so-called *common factors* are well-researched and offer a helpful framework for understanding the therapeutic effects of psychedelics. Results from meta-analyses highlight the centrality of alliance bond experience, agreement on means and goals of the therapeutic endeavor, perceived empathy and positive regard from the therapist/setting, opportunities for emotional expression and experience, activating resources, motivational clarification, and mastery by self-management and emotion regulation (Grawe, 2000; Norcross and Lambert, 2018; Wucherpfennig et al., 2024).

The findings from recent clinical trials, employing psilocybin within supportive therapeutic contexts, suggest sustained antidepressant effects alongside rather minor and temporary adverse events (Watford and Masood, 2024). However, the findings of early trials face methodological challenges concerning blinding and pre-treatment expectancy and must therefore be interpreted with caution (Mertens et al., 2022). Despite challenges, the growing evidence indicates psilocybin might serve as an attractive alternative to traditional drugs for depression (Watford and Masood, 2024). However, to inform new treatment approaches, a comprehensive understanding of the mechanisms underlying the antidepressant effect is necessary. While various theoretical articles focus on distinct mechanisms of psilocybin, a comprehensive review including recent empirical data on both neurobiological and psychological antidepressant effects is lacking.

This systematic review primarily aims to address this knowledge gap by providing a holistic overview, integrating both the neurobiological and psychological effects of psilocybin previously associated with antidepressant mechanisms.

Other levels of explanation, such as molecular and cellular mechanisms, may be crucial as well and have been discussed extensively in previous reviews (e.g., Jaster and González-Maeso, 2023).

Moreover, this review also acknowledges the ongoing debate over whether psilocybin operates solely through direct pharmacological means, a notion which is already questioned for classic drugs for depression (Rief et al., 2016), or if its effects are complemented by subjective experiences, thereby catalyzing common factors of change in psychotherapy (Goodwin et al., 2024; Gründer et al., 2024b; Mander et al., 2013; Mertens et al., 2020; Norcross and Lambert, 2018). As a secondary objective, this review seeks to contribute to this discourse by discussing parallels between its findings and established concepts of psychedelic effects and common factors of psychotherapy. The outcomes may inform future research and add to an empirical base for instruments measuring psychotherapeutic change mechanisms in psychedelic-assisted therapy, such as the General Change Mechanisms Questionnaire (GCMQ) introduced by Wolff et al. (2024).

## Methods

### Protocol

This review was conducted using the preferred reporting items for systematic reviews and meta-analysis protocol (PRISMA; Page et al., 2021) and the guidelines outlined in the Cochrane Handbook for Systematic Reviews (Higgins et al., 2023). Inclusion and exclusion criteria were structured based on the PICOS framework (Population, Intervention, Comparator, Outcome, Study Characteristics). The research protocol has been pre-registered under https://www.osf.io/eswn7.

### Inclusion criteria

The review included studies that met the specified inclusion criteria:

#### Population

Human populations with diagnosed depression or healthy subjects in studies directly related to depression. Samples with depressive conditions included various diagnoses, such as major depressive disorder (MDD) and TRD.

#### Intervention

Studies involving psilocybin therapy, with a minimum dose of 10 mg in the test group, administered in a controlled and guided study context. Microdosing was excluded, as current evidence indicates that significant antidepressant effects are primarily observed at moderate to higher doses in clinical trials (e.g., Goodwin et al., 2022).

#### Comparator

Studies with randomized placebo or active control groups. Alternatively, pre–post comparisons, if higher standard designs are not available on the same issue, for example, follow-up studies.

#### Outcome

Studies focusing on antidepressant neurological or psychological mechanisms of psilocybin and associations with common factors of psychotherapy.

#### Study characteristics

Clinical trials of all types (quantitative and qualitative) were published from 2015 onwards in English or German language. This review included studies from 2015 onwards to focus on modern psilocybin trials with controlled protocols, depressive samples, and recent advancements in neuroimaging and brain functioning research.

### Exclusion criteria

Studies were excluded if they:

(a) Administered other psychedelic substances than psilocybin. Although comparable reviews, such as (Gattuso et al., 2023), often include other serotonergic hallucinogens like lysergic acid diethylamide (LSD) or dimethyltryptamine (DMT), this review focuses on psilocybin due to its recent prominence in research and clinical trials. In addition, it may be beneficial to investigate the effects of one substance in isolation during the early stages of this research field and later generalize and compare it to other related substances. It is also important to note, that although LSD and DMT are also classified as classic serotonergic psychedelics, their drug effects differ in duration and intensity from those of psilocybin (Gattuso et al., 2023);
(b) Used retrospective data on past dosing sessions that were not controlled parts of the study;
(c) Investigated clinical benefits without the underlying mechanisms responsible for the effect. Studies focusing primarily on quantifying clinical outcomes, such as antidepressant efficacy, without exploring the mechanisms driving these effects were excluded, as this review aimed to explore potential antidepressant neurobiological and psychological mechanisms of psilocybin;
(d) Focused on fundamental research on brain mechanics and subjective effects of psilocybin lacking a clear connection to depression symptoms or antidepressant effects; and
(e) Were review articles and conceptual or theoretical articles.

### Search strategy and identification of studies

Between November 18, 2023 and December 5, 2023, a comprehensive literature search was conducted using multiple online databases including PubMed, PsycINFO, PsycARTICLES, Web of Science, and Clinicaltrials.gov. Search terms were identified upon existing literature on antidepressant neural and psychological mechanisms of psilocybin (i.e., FC, neural plasticity, flexibility, openness, connectedness, experiential avoidance) and common factors of psychotherapy or terms serving as proxies (i.e., alliance, empathy, problem activation, resource activation, emotional activation) and combined with “psilocybin.” An exhaustive list of search terms and operators is available in the Supplemental Material.

### Study selection

The search results were imported into *Covidence*, a web-based collaboration software platform for systematic reviews (Covidence, 2023). Duplicates were removed automatically by Covidence and references were screened for eligibility by the author J.L. in two rounds: (1) title and abstract screening followed by (2) full-text review.

### Data extraction

Data were extracted using a predefined template that included the aim of the study, sample characteristics such as clinical status, age, gender, sample size, information on treatment including study design, conditions, dose of psilocybin/placebo, information on supportive therapy, and outcome measures. To address the interdisciplinary nature and the aims of this review, the outcome measures were specified, differentiating between brain dynamics, psychological findings, and secondary outcomes. We decided against conducting a meta-analysis due to the nature of the research question and the insufficient availability of data that could be statistically aggregated. Instead, we chose to perform a narrative synthesis.

### Risk of bias assessment

The included studies were evaluated for their risk of bias and study quality. Randomized controlled trials (RCT) were assessed using the revised Cochrane Risk-of-Bias tool for randomized trials (RoB 2; (Sterne et al., 2019). The overall risk of bias was assessed on a scale with three tiers: low risk, some concerns, and high risk. Non-RCTs were assessed using the Risk Of Bias In Non-randomized Studies—of Interventions tool (ROBINS-I; (Sterne et al., 2016). The overall risk of bias was assessed on a scale with four tiers: low, moderate, serious, and critical. Qualitative reports were assessed using the Critical Appraisal Skills Programme (CASP; Critical Appraisal Skills Programme, 2024). All three tools are commonly used in systematic reviews and were recommended in the Cochrane Handbook for systematic reviews (Higgins et al., 2023).

## Results

### Study selection

**Figure 1.**
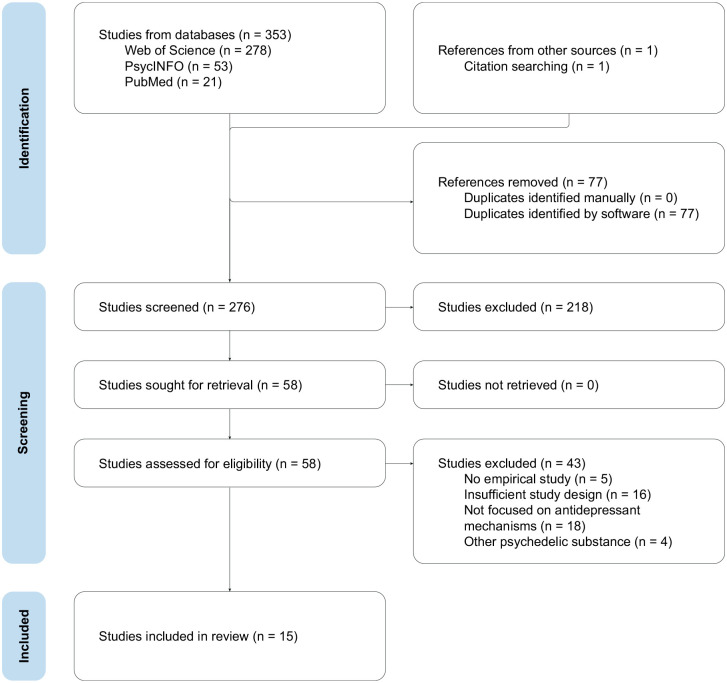
PRISMA flow diagram. PRISMA: preferred reporting items for systematic reviews and meta-analysis.

The search resulted in a total of 352 papers across databases. After the removal of 77 duplicates, 275 studies were screened against title and abstract. Fifty-seven references were assessed as eligible for full-text screening. A citation search of these articles provided one more result that was included in a full-text review, increasing the total count to 58 papers. After a full-text review, 43 studies were excluded. See Figure 1 for a detailed flow chart of the process and all exclusion reasons. A total of 15 studies fulfilled the inclusion criteria and were included in the review.

### Overview of included studies

Table 1 provides an overview of the aims, sample and treatment characteristics, and outcome measures of the 15 included studies. The included studies were based on data from seven distinct trials with a total of 251 probands. The trial ID for each study is referenced in the table under the *Trial* column, indicating when multiple analyses are based on the same trial. For a comprehensive mapping of the included studies and the source trials, please refer to the Supplemental Material. To address the heterogeneity of the included studies and facilitate the differentiation of therapeutic mechanisms, the studies were clustered into four preliminary domains based on their study aims and outcome measures: (1) *Brain dynamics*; (2) *Emotion regulation*; (3) *Cognition & self-referential processing*; and (4) *Connectedness & interpersonal functioning*. Yet, the domains are not exhaustive, and some studies contributed to more than one domain due to their integrative study aims.

**Table 1.** Overview of studies and outcome measures.

Trial	Reference	Aim of study	Sample characteristics				Treatment				Outcome measures	
			Clinical status	Age	Gender	N	Design	Conditions	Dose of psilocybin	Therapy	Brain dynamics & ROI	Psychological measures
Brain dynamics												
1	Carhart-Harris et al. (2017)	Brain mechanics: FC, brain modularity, cerebral blood flow.	TRD	44.7	32% **♀**, 68% ♂	19	Open-label	1. Psilocybin	10 & 25 mg 7 days apart	Yes	CBF, BOLD, RSFC of bilateral PH, vmPFC, sgACC, and bilateral amygdala	Depression (QIDS-SR16)
1, 2	Daws et al. (2022)	Brain functioning: global integration, FC, modularity, and DMN activity.	TRD & MDD	*Open-label*: 44.7, *RCT*: 41	*Open-label*: 32% **♀**, 68% ♂ *RCT*: 34% **♀**, 66% ♂;	*Open-label*: 19, *RCT*: 59	Open-label, RCT	*Open-label*: 1. Psilocybin *RCT*: 1. Psilocybin 2. Escitalopram	*Open-label*: 10 & 25 mg 7 days apart *RCT*: 3 weeks apart 2 × 25 mg & daily placebo or 2 × 1 mg and daily escitalopram	Yes	FC, brain modularity, and network flexibility of DMN, EN, and SN hubs	Depression (BDI)
3	Skosnik et al. (2023)	EEG correlates of neural plasticity.	MDD	42.8	68% **♀**, 32% ♂	19	NRCT	1. Placebo 2. Psilocybin	Placebo, 4 weeks later 0.3 mg/kg psilocybin	Yes	Evoked theta power measured via EEG.	Depression (GRID HAM-D-17)
4	Smigielski et al. (2019a)	DMN FC and self-consciousness.	Healthy	51.7	39% **♀**, 61% ♂	38	RCT	1. Psilocybin 2. Placebo	0.315 mg/kg on 4th day	No	FC of DMN hubs: mPFC, PCC, and bilateral AG	Altered states of consciousness (5D-ASC), Changes in psychosocial domains (PEQ)
Emotion regulation												
1	Mertens et al. (2020)	FC connectivity of amygdala-vmPFC during emotional face processing.	TRD	44.7	32% **♀**, 68% ♂	19	Open-label	1. Psilocybin	10 & 25 mg 7 days apart	Yes	FC in amygdala and vmPFC	Depression (QIDS-SR16 & BDI), Rumination (RRS)
1	Shukuroglou et al. (2023)	Music-evoked emotions and FC.	TRD	44.7	32% **♀**, 68% ♂	19	Open-label	1. Psilocybin	10 & 25 mg 7 days apart	Yes	FC of NAc	Music evoked emotion (GEMS), and pleasure during fMRI (SHAPS & 1–10 rating scale)
2	Zeifman et al. (2023)	Experiential avoidance.	MDD	41	34% **♀**, 66% ♂	59	RCT	1. Psilocybin 2. Escitalopram	3 weeks apart 2 × 25 mg & daily placebo or 2 × 1 mg and daily escitalopram	Yes	n/a	Experiential avoidance (BEAQ), Well-being (WEMWBS), Depression (MADRS & QIDS-SR16), Suicidal ideation (SIDAS), Anxiety (STAI-Trait), Connectedness (WCS), Ego dissolution (EDI), Mystical experience (MEQ), Emotional breakthrough (EBI), Psychological insight (PIQ)
Cognition & self-referential functioning												
2	Weiss et al. (2023)	Big five personality traits.	MDD	41	34% **♀**, 66% ♂	59	RCT	1. Psilocybin 2. Escitalopram	3 weeks apart 2 × 25 mg & daily placebo or 2 × 1 mg and daily escitalopram	Yes	n/a	Big Five Personality (BFI & BFAS), Absorption (MODTAS), Impulsivity (BIS-B) Mystical experience (MEQ), Emotional Breakthrough (EBI), all on a scale of 0–100: Emotional Insight, Intensity, and Expectancy
4	Smigielski et al. (2019b)	Mindfulness and psychosocial functioning.	Healthy	51.7	41% **♀**, 59% ♂	39	RCT	1. Psilocybin 2. Placebo	0.315 mg/kg or placebo	No, but intense meditation practice.	n/a	Trait mindfulness (FMI), State mindfulness (TMS), Meditation depth (MEDEQ), Altered states of consciousness (5D-ASC), Mystical experiences (M-scale), Changes in behavior and attitudes (LCI-R)
5	Doss et al. (2021)	Cognitive and neural flexibility and FC.	MDD	39.8	67% **♀**, 33% ♂	24	RCT	1. Psilocybin 2. Control + Delayed Psilocybin	2 doses: 20 mg/70 kg and 30 mg/70 kg ~ 1.6 weeks apart or placebo	Yes	Neural flexibility (dynamics of FC), RSFC of ACC and PCC	Depression (GRID-HAM-D-17) Executive function (PCET)
6	Mason et al. (2021)	Creative thinking and biomarkers of changes.	Healthy	23	42% **♀**, 58% ♂	60	RCT	1. Psilocybin 2. Placebo	0.17 mg/kg or placebo	No	FC of DMN hubs: mPFC and hippocampus	Convergent and divergent thinking (PCT & AUT), Altered states of consciousness (5D-ASC)
Connectedness & interpersonal functioning												
1	Watts et al. (2017)	Qualitative accounts of antidepressant mechanisms and experiences of treatment with psilocybin.	TRD	44.7	32% **♀**, 68% ♂	19	Open-label	1. Psilocybin	10 & 25 mg 7 days apart	Yes	n/a	Qualitative structured interviews on patient experiences
2	Watts et al. (2022)	Connectedness (self, others, world) construct.	MDD	41	34% **♀**, 66% ♂	59	RCT	1. Psilocybin 2. Escitalopram	3 weeks apart 2 × 25 mg & daily placebo or 2 × 1 mg and daily escitalopram	Yes	n/a	Depression (QIDS-SR16), Connectedness (WCS)
2	Murphy et al. (2022)	Therapeutic alliance, psychedelic experience, and treatment outcomes.	MDD	41	34% **♀**, 66% ♂	59	RCT	1. Psilocybin 2. Escitalopram	3 weeks apart 2 × 25 mg & daily placebo or 2 × 1 mg and daily escitalopram	Yes	n/a	Depression (QIDS), Therapeutic relationship (STAR-P), Rapport: 0–100 scale, Mystical experience (MEQ), Emotional Breakthrough (EBI)
7	Pokorny et al. (2017)	Empathy and moral decision-making.	Healthy	26.7	47% **♀**, 53% ♂	32	RCT	1. Psilocybin 2. Placebo	0.215 mg/kg, placebo (mannitol) 10 days apart	No	n/a	Empathy (MET), Moral dilemma (MDT), Altered states of consciousness (5D-ASC), Affect (PANAS)

*Note.* Trial: Source trials; 1 = Carhart-Harris et al. (2017); 2 = Carhart-Harris et al. (2021); 3 = Skosnik et al. (2023); 4 = Smigielski et al. (2019b); 5 = Davis et al. (2021); 6 = Mason et al. (2020); 7 = Pokorny et al. (2017).

*Clinical status*: MDD: major depressive disorder; TRD: treatment-resistant depression; *Design*: NRCT: non-randomized controlled trial; RCT: randomized controlled trial, all RCTs were double-blinded; *Supportive Therapy*: Yes: Preparation sessions before, guiding during dosing, and integration sessions after; *Brain dynamics & ROI*: ACC: anterior cingulate cortex; AG: angular gyrus; BOLD: blood oxygen dependent; CBF: cerebral blood flow; DMN: default mode network; EEG: electroencephalography; EN: executive network; FC: functional connectivity; All measured via fMRI: functional magnetic resonance imaging unless otherwise specified; mPFC: medial prefrontal cortex; NAc: nucleus accumbens; PCC: posterior cingulate cortex; PH: parahippocampal gyrus; ROI: region of interest; RSFC: resting-state functional connectivity; sgACC: subgenual anterior cingulate cortex; SN: salience network; vmPFC: ventromedial prefrontal cortex; *Psychological Measures*: 5D-ASC: five dimensional altered states of consciousness scale; AUT: alternative use task; BDI: Beck depression inventory; BEAQ: brief experiential avoidance questionnaire; BFAS: big five aspects scale; BFI: big five inventory; BIS-B: Barret impulsivity scale-brief; EBI: emotional breakthrough inventory; EDI: ego dissolution inventory; FMI: Freiburg mindfulness inventory; GEMS: Geneva emotional music scale; GRID HAM-D-17: Hamilton rating scale for depression; LCI-R: life changes inventory-revised; M-scale: mysticism scale; MADRS: Montgomery-Åsberg depression rating scale; MDT: moral distress thermometer; MEDEQ: meditation depth questionnaire; MET: multifaceted empathy test; MEQ: mystical experience questionnaire; MODTAS: modified-Tellegen absorption scale; PANAS: positive and negative affect schedule; PCET: Penn conditional exclusion test; PCT: picture concept task; PEQ: persisting effects questionnaire; PIQ: psychological insight questionnaire; QIDS-SR16: quick inventory of depressive symptomatology; RRS: rumination response scale; SHAPS: Snaith-Hamilton pleasure scale; SIDAS: suicidal ideation attributes scale; STAI-Trait: state-trait anxiety inventory; STAR-P: scale to assess the therapeutic relationship; TMS: Toronto mindfulness scale; WCS: Watts connectedness scale; WEMWBS: Warwick-Edinburgh mental well-being scale; Qualitative structured interviews on patient experiences: for example, What was your experience of depression before the study? What treatments have you tried? What happened during dosing? How did you feel in the weeks and months afterward? How does it compare to other treatments?

#### Clinical status

Seven studies utilized data from a trial with a sample of individuals diagnosed with MDD and five studies included samples of probands with TRD. In addition, four studies used samples of healthy probands, aligning with depression-orientated study aims. One study included two trials with samples of MDD and TRD, respectively.

#### Study design

Five studies were single-arm open-label designs with pre–post measures. Among these, one study utilized qualitative methods, while the remaining four employed quantitative methods. Ten studies used RCT and one study used data from a non-randomized, but controlled trial (NRCT). One study included both, an open-label trial and an RCT. The RCTs either used a placebo or the serotonin reuptake inhibitor *escitalopram* as an active comparator.

#### Dose of psilocybin

The administered dose of psilocybin ranged from 0.17 to 0.315 mg/kg body weight or up to 25 mg in max dose independent of body weight. Probands received either one or two doses across all studies. In all cases, psilocybin was administered orally.

#### Supportive therapy

All studies with clinical samples included supportive therapy sessions before, during, and after dosing sessions. Preparation sessions were commonly used for psychoeducation and intention-building. Therapeutic guidance during the dosing sessions had a non-directive, supportive character, and the sessions after the dosing were used for integration work. Yet, the supportive sessions varied in frequency, skill level of the therapist, and specific therapeutic concepts employed. Probands of studies without supportive therapy were nonetheless briefed intensely and guided by health practitioners throughout the entire trial.

#### Outcome measures

The outcome measures of the included studies were heterogeneous. For better clarity, (1) brain dynamics measures and region of interest, (2) psychological measures, and (3) secondary outcomes were reported separately. Eight studies employed brain dynamic measures including FC, cerebral blood flow, or blood oxygenation level-dependent via functional magnetic resonance imaging (fMRI) or neural plasticity via electroencephalography (EEG). All 15 included studies employed psychological measures. The type of measure was diverse, ranging from depression severity to assessments of personality, creativity, emotion regulation, acute psychedelic effect, therapeutic relationship, connectedness, anxiety, rumination, empathy, and measures of moral decision-making.

### Outcomes

Table 2 contains a narrative synthesis of outcomes grouped by potential mechanisms of action investigated. Due to the heterogeneity in the quality of study design and the aims of included studies, the reported outcomes were dependent on the best available body of evidence. Statistical significance was considered at a *p*-value below 0.05 (*p < 0.05; **p < 0.01; ***p < 0.001). The studies reported different effect sizes including correlation coefficients *r*, Cohen’s *d*, Hedge’s *g*, and $\eta^{2}$ /partial $\eta^{2}$ . Adhering to standard conventions, a |*r*| of 0.10 is considered small, 0.30 medium, and 0.50 large; for both *d* and *g* 0.20 is considered small, 0.50 medium, and 0.80 large; while $\eta^{2}$ or partial $\eta^{2}$ of 0.01 is considered small, 0.06 medium and 0.14 large (Cohen, 1988).

**Table 2.** Outcomes and risk of bias assessment of included studies.

Trial	Reference	Follow-up	Brain dynamics outcomes	Psychological outcomes	Secondary outcomes	Risk of bias
Brain dynamics						
1	Carhart-Harris et al. (2017)	5 weeks, 3 months, 6 months	Decreased CBF in the left amygdala, the left Heschl’s gyrus, left precentral gyrus, left planum temporale, left superior temporal gyrus, right supramarginal gyrus, and right parietal operculum 1 day post-psilocybin. Decreased RSFC between parahippocampus and prefrontal cortex. Amygdala RSFC was not altered post-treatment.	Depression severity decreased in 18/19 patients from baseline to 1 week*** (*d* = 2.2), 5 weeks*** (*d* = 2.3) post-treatment and sustained 3 months*** (*d* = 1.5) and 6 months*** (*d* = 1.4) follow-up. 47% fulfilled the criteria for response and 21% fulfilled the criteria for remission at week 5.	CBF decreases in the amygdala correlated with reductions in depressive symptoms pre–post psilocybin** (*r* = 0.59). Decreased PH-PFC RSFC did not correlate with reductions in depressive symptoms pre–post psilocybin. But it relates to treatment response at 5 weeks: responders showed significantly greater PH-PFC RSFC decreases than non-responders*.	ROBINS-I: Moderate
1, 2	Daws et al. (2022)	*Open-label*: 6 months *RCT*: 6 weeks	*Open-label.* Decreased brain modularity* (*d* = 0.72). Decreased within-DMN connectivity** (*d* = 0.75). Increased FC between DMN and EN** (*d* = 0.75) and SN** (*d* = 0.72). *RCT*. Brain network modularity was significantly reduced at 3 weeks post-psilocybin* (*d* = 0.47). In the escitalopram group, network modularity did not change from baseline (*d* = 0.02).	*Open-label*: Compared to baseline, significant reductions in depression symptoms at 1 week*** (*d* = 1.78) and at 6 months*** (*d* = 1.07). *RCT*. Significant differences in depression severity between psilocybin and escitalopram at 2 weeks** (*d* = 0.98), 4 weeks* (*d* = 0.77), and at 6 weeks* (*d* = 0.75), relative to baseline, all in favor of the psilocybin group.	*Open-label.* Decreased brain modularity correlated with improvement in BDI scores at 6 months post-treatment* (*r* = 0.64). *RCT*. Significantly reduced brain network modularity correlated with improvements in depression symptom severity at 3 weeks post-psilocybin* (*r* = 0.42) but not post-escitalopram. Exploratory analysis revealed increased executive network dynamic flexibility correlated with symptom improvement at 3 weeks post-psilocybin** (*r* = −0.76) but not post-escitalopram.	*Open-label.* ROBINS-I: Moderate *RCT.* RoB 2: Some concerns
3	Skosnik et al. (2023)	2 weeks	No difference in average theta power amplitudes between placebo and psilocybin 24 h post-treatment. 2 weeks post-treatment, average theta power amplitudes doubled after psilocybin, but not placebo (*p* = 0.09).	Significant effect of time*** and drug*** on GRID-HAM-D-17 scores at 2 weeks post-treatment in favor of psilocybin.	Significant negative correlation between the change in depression scores and change in theta power 2 weeks post-psilocybin* (Spearman’s rho = −0.57). Decreases in depression symptoms were associated with increases in theta amplitudes at 2 weeks post-psilocybin.	ROBINS-I: Moderate
4	Smigielski et al. (2019a)	4 months	Increased RSFC within antero-ventral DMN including ACC* in the psilocybin group. Decreased FC between mPFC and: PCC** right AG* and left AG*, during open awareness meditation in the psilocybin, but not in the placebo group.	Higher mean global positive change score of the PEQ scores in the treatment group after 4 months compared to control***. This effect was positively correlated with acute oceanic self-boundlessness scores (5D-ASC) during psilocybin intake** (*r* = 0.66).	Negative correlation between oceanic self-boundlessness and pre-post change in FC between the mPFC and PCC* (*r* = 0.595). Increases in mPFC-PCC RSFC predicted a positive change in attitudes (5D-ASC) 4 months post psilocybin treatment*. A decrease in mPFC-AG connectivity during focused attention meditation was also predictive of that outcome*.	RoB 2: Some concerns
Emotion regulation						
1	Mertens et al. (2020)	3 months	Decreased FC between the vmPFC and right amygdala during emotional face processing 1 day post-psilocybin. Increased FC and responsiveness within the vmPFC and bilateral amygdala with occipital-parietal cortices during emotional face processing 1 day post-psilocybin.	Rumination levels were significantly reduced between baseline at 1 week*** and 3 months post-psilocybin***.	Decreased FC between the vmPFC and right amygdala during emotional face processing 1 day post-psilocybin did not correlate with depression levels at 1 week, but did correlate to reduced rumination scores 1 week post-psilocybin* (*r* = 0.54).	ROBINS-I: Moderate
1	Shukuroglou et al. (2023)	3 months	Decreased FC of the NAc with DMN post-treatment compared to pre-treatment during music scans versus no music scans.	Significant increase of pleasure scores during music scans post-psilocybin*** and significant increases in the music-evoked Peacefulness* post-treatment. Simultaneous post-treatment decrease of music-evoked Sadness*. Anhedonia scores significantly decreased from baseline to 1 day***, 1 week***, and 3 months** post-psilocybin.	Music-evoked pleasure post-psilocybin was associated with significantly greater decreases of anhedonia scores at 1 week post-treatment* (*r* = −0.52). Decreased NAc-DMN FC post-treatment compared to pre-treatment during music scans versus no music scans, was not associated with the anhedonia effects.	ROBINS-I: Moderate
2	Zeifman et al. (2023)	6 weeks	n/a	Significant reduction of experiential avoidance pre-6 weeks follow-up after psilocybin***, but not after escitalopram. Increases in well-being*** and decreases in clinician-assessed depression severity**, self-reported depression severity*, suicidal ideation**, and trait anxiety** at 6 weeks post-treatment were significantly mediated through reductions in experiential avoidance in the psilocybin group, but not in the escitalopram group. In the psilocybin group, reductions in experiential avoidance were positively correlated with experiences of ego dissolution** (*r* = -0.48) and psychological insight* (*r* = −0.38).	Improvements in mental health via reductions in experiential avoidance were found to be significantly serially mediated by increases in connectedness for increases in well-being*, as well as decreases in clinician-assessed depression severity*, self-reported depression severity**, and trait anxiety* in the psilocybin, but not the escitalopram group.	RoB 2: Some concerns
Cognition & self-referential processing						
2	Weiss et al. (2023)	6 weeks & 6 months	n/a	At 6 weeks, Neuroticism***, Introversion***, Disagreeableness***, and Impulsivity*** were significantly decreased, and Openness* and Absorption*** were significantly increased in the psilocybin condition. After 6 months, Neuroticism** and Disagreeableness*** remained decreased from baseline levels. The escitalopram condition induced similar effects, except for Absorption and Introversion, remaining unchanged. No significant between-condition effects.	Positive pre-treatment expectancy for escitalopram showed a significant moderation effect for Neuroticism** and Conscientiousness** in the escitalopram condition. If expectancy was set to zero in a counterfactual model, the change of Neuroticism and Conscientiousness lost significance in the escitalopram condition. Expectancy did not moderate outcomes in the psilocybin condition.	RoB 2: Some concerns
4	Smigielski et al. (2019b)	4 months	n/a	Post-retreat trait mindfulness score was higher following psilocybin compared with placebo***. Meditation depth was greater in the psilocybin group than in the placebo group on the dosing day* and across time***. Following psilocybin, ratings on: Oceanic Boundlessness***, Unity**, Visionary Restructuralization***, and Vigilance Reduction* were higher compared to placebo. At 4-month follow-up, perceived changes in behavior and attitudes were significantly higher in the psilocybin arm than in the placebo arm***. Self-rated scores on the LCI-R subscales Appreciation of Life, Self-Acceptance, Quest for Meaning/Sense of Purpose, and Appreciation of Death were all significantly higher in the psilocybin group compared with placebo**.	Psilocybin intake* (η2 = 0.15) and loss of ego boundaries** (η^2^ = 0.20) explained a significant amount of variance in total LCI-R score. Experience of unity*** (η^2^ = 0.32) explained a significant amount of variance in score on the Self-Acceptance scale.	RoB 2: Some concerns
5	Doss et al. (2021)	2 months	Across the brain RSFC decreased and FC increased after psilocybin. No change in ACC-PCC RSFC, but a significant increase in ACC-PCC FC between 1 week post psilocybin** (*d* = 0.64).	Immediate treatment with psilocybin showed significantly lower depression scores at weeks 1 and 4 than the delayed treatment group at comparable time points at weeks 5** (*d* = 2.5) and week 8** (*d* = 2.6). 71% of participants fulfilled criteria for response and 54% and 58% fulfilled criteria for remission at week 1 and 4. Perseverative errors on the PCET decreased from baseline to 1 and 4 weeks post-psilocybin***. Perseverative errors did not change in the delayed group prior to psilocybin intake.	Positive correlation between increases in FC and decreases in PCET perseverative errors 1 week post* (*r* = 0.48). Indications for a nuanced relationship (inverted u-shaped) between cognitive and neural flexibility. Changes in depression scores were not associated with changes in perseverative errors.	RoB 2: Some concerns
6	Mason et al. (2021)	1 week	Within anterior and posterior DMN, significantly less coactivation following psilocybin compared to placebo. In between-network FC was increased after psilocybin relative to placebo. Higher FC between anterior and posterior DMN and the frontoparietal control network and between the anterior DMN and the salience network.	Psilocybin significantly increased all domains of the 5D-ASC, including insightfulness compared to placebo*** (*d* = 1.78) pre-1 week post. Psilocybin acutely decreased convergent thinking** (*d* = 0.85) and aspects of divergent thinking, fluency** (*d* = 0.84), and originality* (*d* = 0.65) compared with placebo. At 1 week post-treatment convergent thinking was still significantly decreased* (*d* = 0.60). Measures of divergent thinking showed a change in trend at 1-week follow-up. They either matched the level of placebo condition (fluency) or significantly improved compared to placebo* (novelty; (*d* = 0.52).	Exploratory analysis indicates that decreases in FC within DMN under psilocybin predicted an acute decrease in scores of originality and increased insightfulness along with increased long-term idea novelty. It further suggests that higher levels of between-network FC between DMN and FPN were the strongest predictor of larger negative changes in acute and long-term convergent thinking.	RoB 2: Some concerns
Connectedness & interpersonal functioning						
1	Watts et al. (2017)	6 months	n/a	17/19 patients described a change from a depressive state of disconnection from the self, others, and the world toward experiences of connectedness to these qualities. 16/19 patients reported a change from avoidance of difficult or painful emotions and memories toward greater motivation and capacity for acceptance and confrontation. Patients shared that, even if feeling the previously avoided emotions was very intense (16/19), they experienced relief and bliss by surrendering to the emotion (13/19). 15/19 reported that their emotional repertoire expanded long term after treatment.	16/19 patients reported that traditional treatment of depression, despite temporary benefits, reinforced disconnection and avoidance (e.g., by emotional blundering of antidepressants), whereas treatment with psilocybin promoted confrontation and lasting reconnection.	CASP: 8/9
2	Watts et al. (2022)	6 weeks	n/a	Greater WCS scores at 6 weeks following psilocybin treatment than escitalopram** (η*p*^2^ = 0.133), while groups did not differ at baseline. No significant change in WCS scores for both psilocybin non-responders and escitalopram non-responders. Significant increase of WCS scores for both psilocybin responders*** (Hedge’s *g* = 2.29) and escitalopram responders** (Hedge’s *g* = 0.95) pre-6 weeks post. WCS scores were significantly higher at 6 weeks for psilocybin responders compared with escitalopram responders*** (Hedge’s *g* = 1.52) but not higher for psilocybin non-responders than escitalopram non-responders.		ROBINS-I: Moderate
2	Murphy et al. (2022)	6 weeks	n/a	Strong therapeutic alliance significantly predicted depression severity at 6 weeks via increases in pre-session rapport***, stronger emotional breakthrough ***, and mystical-type experience** in the psilocybin group. In the escitalopram condition, acute experiences and final outcomes were not predicted by the therapeutic relationship. Therapeutic alliance before the second session was positively impacted by emotional breakthrough***, but not mystical experience, during the first psilocybin dosing session.	Depression severity at week 6 was more strongly affected by emotional breakthrough in the first, and mystical experience in the second dosing session.	RoB 2: Some concerns
7	Pokorny et al. (2017)	n/a	n/a	Implicit* and explicit emotional empathy** increased in the psilocybin group compared to the placebo, whereas cognitive empathy showed no significant change. Ratings on the positive affect scale* of PANAS were significantly increased following psilocybin, but not placebo. Negative affect ratings remained unchanged in both groups. Psilocybin did not affect moral decision-making compared to placebo.	The increase in implicit emotional empathy was significantly predicted by changed meaning of percepts**, an acute effect of psilocybin measured by 5D-ASC.	RoB 2: Some concerns

*Note*. *p < 0.05; **p < 0.01; ***p < 0.001.

Response = ⩽ 50% reductions in QIDS-SR16, BDI score or GRID-HAMD-17 score; Remission = BDI score ⩽ 9 or GRID-HAMD-17 score ⩽ 7; Risk of Bias = overall risk of bias assessment conducted for RCTs via the Revised Cochrane risk-of-bias tool for randomized trials (RoB 2), for NRCTs via The Risk Of Bias In Non-randomized Studies—of Interventions (ROBINS-I) assessment tool, and for qualitative studies using the CASP.

5D-ASC: five dimensional altered states of consciousness scale; ACC: anterior cingulate cortex; BDI: Beck depression inventory; CASP; Critical Appraisal Skills Programme; CBF: cerebral blood flow; DMN: default mode network; EN: executive network; FC: functional connectivity; GRID HAM-D-17: Hamilton rating scale for depression; mPFC: medial prefrontal cortex; NAc: nucleus accumbens; PANAS: positive and negative affect schedule; PCC: posterior cingulate cortex; PH: parahippocampal gyrus; RCT: randomized controlled trials; RSFC: resting state functional connectivity; SN: salience network; vmPFC: ventromedial prefrontal cortex; WCS: Watts connectedness scale.

#### Brain dynamics

A total of eight studies examined the sub-acute effects of psilocybin on brain dynamics based on data from one open-label trial and five RCTs. The most investigated subjects were alterations in FC of brain regions associated with depression via fMRI. Hubs of the DMN, such as the mPFC, vmPFC, PCC, and AG were investigated as key regions of interest along with the ACC, amygdala, PH, and NAc.

##### Functional connectivity

All studies found alterations in FC following psilocybin compared to placebo or baseline. The predominant finding observed in four studies was a reduction in resting-state connectivity within the DMN, coupled with a simultaneous increase in FC between the DMN and other networks such as the executive network (EN), salience network (SN), and visual cortices (Daws et al., 2022; Doss et al., 2021; Mason et al., 2021; Mertens et al., 2020). The study by Smigielski et al. (2019a) on a healthy test group reported a reduction in FC within the DMN correlated with higher scores on measures of oceanic-self-boundlessness, an acute effect of psilocybin linked to feelings of unity and connectedness. In addition, the same study found increased resting-state connectivity within specific DMN hubs, (mPFC-PCC) and decreased FC within other DMN hubs (mPFC-AG) during meditation. Both effects predicted a positive change in psychosocial attitudes at a 4-month follow-up.

While the FC between the DMN and other networks like EN, SN, or visual cortices increased (Daws et al., 2022; Mason et al., 2021; Mertens et al., 2020), it did not universally increase across all brain regions. Mertens et al. (2020) reported a decrease in FC between the vmPFC, a key hub of the DMN, and the amygdala during emotional face processing following psilocybin. This shift was significantly correlated with lower rumination scores 1 week post-psilocybin. Carhart-Harris et al. (2017) found decreased resting state connectivity between the PFC and the parahippocampal gyrus (PH) post-psilocybin, correlating with depression treatment response. Responders showed significantly greater decreases in PFC-PH resting state connectivity compared to non-responders.

##### Global modularity

A two-trial study by Daws et al. (2022) reported a significant decrease in global brain modularity and greater global brain network integration compared to baseline (open-label), and escitalopram (RCT), respectively. This effect significantly correlated with improvements in depression symptoms at 3 weeks following psilocybin, but not escitalopram, and at 6 months compared to baseline. In addition, two studies identified simultaneous global increases in FC across the brain, compared to control and placebo (Doss et al., 2021; Mason et al., 2021).

##### Cerebral blood flow

Carhart-Harris et al. (2017) found a decrease in CBF in multiple brain regions, including the left amygdala, post psilocybin. The decrease in the amygdala correlated with decreased depression symptoms compared with baseline.

##### Neural plasticity

One study investigated neural plasticity by measuring average theta power amplitudes through EEG, serving as a neural correlate, following psilocybin compared to placebo (Skosnik et al., 2023). While the average theta power amplitudes did not change 24 h after psilocybin, it doubled, compared to placebo, 2 weeks post-treatment, indicating enhanced neural plasticity processes and correlating with improvements in depressive symptom severity scores.

#### Emotion regulation

A total of three studies employed measures that were associated with emotion regulation. Two studies used data from an open-label trial that incorporated assessments of brain dynamics. One study was based on an RCT solely with psychological outcomes. Across studies, two main effects on emotion regulation have formed following psilocybin treatment in supportive contexts. First, a significant decoupling occurred between limbic brain regions and the DMN during exposure to emotional stimuli, concurrently accompanied by strengthened connectivity to visual cortices. Second, patients were more open, non-judgmental, and accepting toward challenging or distressing emotions and sensations, demonstrating less experiential avoidance.

##### Altered connectivity of limbic regions

Two studies investigated altered brain dynamics following psilocybin during exposure to emotional stimuli including emotional faces and emotional music (Mertens et al., 2020; Shukuroglou et al., 2023). Both reported decreased FC between limbic regions including amygdala and NAc and the DMN. Moreover, a simultaneous increase in FC of both amygdala and DMN with occipital-parietal-cortices was reported, suggesting a heightened influence of external visual stimuli during the processing of emotions and self (Mertens et al., 2020).

##### Anhedonia

One study reported significant increases in pleasure and peacefulness listening to music as well as reduced music-evoked sadness post-psilocybin compared to baseline (Shukuroglou et al., 2023). In the same study, anhedonia scores improved significantly from baseline to 1 day, 1 week, and 3 months post-treatment. While the decoupling of DMN and NAc did not correlate significantly with decreases in anhedonia, post-psilocybin music-evoked pleasure was significantly associated with greater reductions in anhedonia scores at 1 week post-treatment.

##### Experiential avoidance

Experiential avoidance was significantly reduced 6 weeks following psilocybin, but not escitalopram (Zeifman et al., 2023). In a study on qualitative reports of patients’ experiences of treatment with psilocybin, changes in experiential avoidance were among the most common themes (Watts et al., 2017). Sixteen out of nineteen patients reported a change from avoidance of difficult or painful emotions and memories toward greater motivation and capacity for acceptance and confrontation. Patients shared that, even if feeling the previously avoided emotions was very intense, they experienced relief and bliss by surrendering to the emotion. Fifteen out of nineteen reported that their emotional repertoire expanded long term after treatment.

Increases in well-being and decreases in depression severity, suicidal ideation, and trait anxiety at 6 weeks post-treatment were significantly mediated through reductions in experiential avoidance in the psilocybin group, but not in the escitalopram group (Zeifman et al., 2023). All improvements driven by reductions in experiential avoidance were found to be significantly serially mediated by increases in connectedness. Suicidal ideation was the only exception to this pattern. Moreover, the reduction in experiential avoidance was significantly positively correlated with experiences of ego dissolution and psychological insight.

#### Cognition & self-referential processing

Four studies investigated the effect of psilocybin on cognition and self-referential processes, based on data from four RCT trials. Two studies also included measures of brain dynamics to investigate the neural correlates of psilocybin-induced changes to cognitive processes. The investigated psilocybin-induced effects were diverse and included changes in personality, consciousness, mindfulness and meditation depth, and neural and cognitive flexibility.

##### Personality changes

Psilocybin induced significant reductions in Neuroticism, Introversion, Disagreeableness, and Impulsivity, along with increased Openness and Absorption at 6 weeks, persisting for Neuroticism and Disagreeableness at 6 months (Weiss et al., 2023). While neither Introversion nor Absorption changed in the escitalopram control, comparable changes were noted at 6 weeks, with only Neuroticism persisting after 6 months. No significant differences in personality changes were observed between conditions. Pre-treatment expectancy significantly moderated changes in Neuroticism and Consciousness in the escitalopram condition. Expectancy was not shown to significantly moderate any outcomes in the psilocybin condition.

##### Consciousness and self

Psilocybin led to higher post-retreat trait mindfulness scores and greater meditation depth compared to placebo (Smigielski et al., 2019b). In addition, ratings for oceanic boundlessness, visionary restructuralization, and vigilance reduction were significantly higher following psilocybin compared to placebo. At 4 months, perceived changes in psychosocial behavior and attitudes were notably higher in the psilocybin group, along with higher ratings on Self-Acceptance, Appreciation of Life and Death, and Quest for Meaning/Sense of Purpose scales, all surpassing placebo levels. Experience of unity explained a significant amount of variance in score on the subscale Self-Acceptance. See Table 2 for more details.

##### Neural and cognitive flexibility

As previously mentioned, psilocybin induced significant reductions in resting-state brain modularity and increases in between-network FC, indicating greater neural flexibility (Doss et al., 2021). Studies investigating the relationship between neural and cognitive flexibility reported mixed findings. On one hand, psilocybin led to decreased perseverative errors in an executive functions measure indicating improved cognitive flexibility (Doss et al., 2021). On the other hand, psilocybin acutely impaired measures of convergent thinking and aspects of divergent thinking compared to placebo (Mason et al., 2021). While convergent thinking remained decreased at 1 week post-treatment, measures of divergent thinking either matched placebo levels, for example, fluency, or significantly improved compared to placebo, for example, idea novelty at 1-week follow-up. Both studies reported that moderate increases in DMN and global between-network connectivity were beneficial for cognitive functioning, whereas high levels impaired cognitive functioning. While decreases in within-DMN FC were associated with reduced idea originality, they also correlated with increased insightfulness and long-term idea novelty, further underscoring complex effects (Mason et al., 2021). Changes in cognitive flexibility were not associated with changes in depression severity (Doss et al., 2021).

#### Connectedness & interpersonal functioning

Four studies investigated the effect of psilocybin on connectedness and interpersonal functioning, based on two RCTs and one open-label trial. The investigated outcomes included psilocybin-induced effects on connectedness, patients’ qualitative accounts of therapeutic mechanisms, therapeutic alliance, and empathy.

##### Connectedness

In an RCT study by Watts et al. (2022), connectedness was significantly increased at 6 weeks post-treatment following psilocybin compared to escitalopram. Thematic analysis of qualitative patient reports identified increases in connectedness as the most common theme of patients’ experiences of treatment with psilocybin (Watts et al., 2017). In all, 17 out of 19 patients in the qualitative study experienced a change from a depressive state of disconnection from the self, others, and the outside world toward experiences of connectedness with these aspects. This transition was commonly described as the most central antidepressant effect of treatment with psilocybin within the qualitative reports. As previously mentioned, improvements in depressive symptoms that were associated with reduced experiential avoidance were serially mediated by increases in connectedness post psilocybin, but not escitalopram (Zeifman et al., 2023).

##### Interpersonal functioning

Across studies, psilocybin improved various aspects of interpersonal functioning such as rapport and empathy compared to escitalopram and placebo. In addition, a stronger therapeutic alliance was associated with increases in rapport, emotional break-through, mystical-type experiences, and reduced depression severity following psilocybin but not escitalopram (Murphy et al., 2022). We include therapeutic alliance as a proxy of interpersonal functioning, as it encompasses stable interpersonal characteristics of both patients and therapists (Zilcha-Mano and Fisher, 2022). Moreover, psilocybin induced increases in implicit and explicit emotional empathy, while cognitive empathy and moral decision-making remained unaffected compared to placebo (Pokorny et al., 2017).

### Risk of bias assessment

Overall, the included studies were of acceptable quality with moderate risk of bias assessments (see Table 2). All 10 studies based on RCTs were evaluated with some concerns for risk of bias. The main sources of risk of bias were deviations from the intended intervention due to blinding problems and treatment expectancy, both commonly known in psilocybin studies. All five studies based on open-label trials were evaluated with a moderate risk of bias. The main sources for risk of bias were confounding variables not controlled for in baseline measures or control groups. The qualitative study fulfilled 9/10 checklist questions for study quality and can therefore be considered to have low to moderate risk of bias. For a comprehensive risk of bias assessment of all studies, please refer to the Supplemental Material.

## Discussion

This systematic review aimed to add to a holistic understanding of how psilocybin facilitates therapeutic change processes. Consequently, 15 studies of 7 distinct trials have been systematically reviewed, exploring various aspects of candidates for therapeutic change principles, including brain dynamics, emotion regulation, cognition & self-referential processing, and connectedness & interpersonal functioning.

### Brain dynamics

Overall, the included studies reported complex and multi-directional alterations of brain dynamics linked to depression including FC, cerebral blood flow, and neural plasticity. Alterations in FC include key neural circuits such as the cortico-striatal-thalamo-cortical (CSTC) and cortico-claustro-cortical (CCC) pathways that are previously found to play crucial roles in mood regulation, cognitive flexibility, and consciousness (Doss et al., 2022; Vollenweider and Preller, 2020). The predominant finding was a decrease in global modularity and resting state connectivity within DMN hubs and an increase in FC between DMN hubs and other networks, such as the EN or the SN and occipital parietal cortices.

Depression has consistently been associated with changes in DMN FC (Borserio et al., 2021; Yan et al., 2019). Recent reviews link depression with elevated FC within the DMN and with reduced FC of DMN to other brain networks, promoting greater brain modularity (Yan et al., 2019; Zhou et al., 2020). This shift appears to promote rigid brain functioning, associated with depressive self-referential thinking (Zhou et al., 2020). This aligns with other symptoms of depression, such as anhedonia and decreased sensitivity to sensory and emotional information. Previous research found that the CSTC circuit’s dysregulation has been implicated in promoting such rigidity, contributing to persistent negative thought patterns and compulsive behaviors characteristic of depression (Onofrj et al., 2023).

In this review, psilocybin consistently changed the DMN connectivity with numerous clinical improvements. Psilocybin’s modulation of the CSTC circuit may facilitate these changes, as it can reduce overactivity in this loop, thereby decreasing rigid thought patterns and enhancing cognitive flexibility. Less within-DMN connectivity and reduced global brain modularity correlated with greater feelings of unity, connectedness, and improvements in depression severity (Daws et al., 2022; Smigielski et al., 2019b). Psilocybin induced increases in connectivity between the DMN and the EN, SN, and visual cortices, suggesting more adaptive and flexible brain functioning (Daws et al., 2022). However, the connectivity of the DMN to other brain regions was not increased universally. One study found decreased connectivity between the PFC, a key hub of the DMN, and the PH at rest, correlating with reduced depression severity (Carhart-Harris et al., 2017; Mertens et al., 2020). This aligns with previous findings of elevated PFC-PH RSFC in depression (Kaiser et al., 2015). Another study of this review found decreases in PFC-amygdala FC during emotional face processing, correlating with reduced rumination (Mertens et al., 2020). This phenomenon may also involve the CCC circuit, where psilocybin alters the CCC interactions, leading to changes in sensory integration and emotional processing, thereby enhancing bottom-up sensory input during emotional experiences. This clinical improvement challenges the common notion that reduced PFC-amygdala FC is usually linked to depression (Kaiser et al., 2015).

We interpret the reduced prefrontal coactivation as indications for decreased inhibitory top-down input from the PFC to the amygdala, consequently resulting in heightened amygdala responsiveness (Mertens et al., 2020). A recent study by Stoliker et al. (2024) further supports the decreased top-down input from resting state networks involved in cognition, including the DMN, to the amygdala post-psilocybin. Notably, Stoliker and colleagues also reported heightened connectivity of the amygdala with the central EN, indicating a complex, nuanced relationship. In addition, Mertens et al. (2020) found increases in FC of the amygdala to visual cortices, suggesting greater importance of sensory bottom-up input during emotional processing.

Although preliminary due to insufficient comparability of clinical and healthy samples, these findings imply greater amygdala responsiveness and greater importance of sensory information during emotional processing post-psilocybin. This notion resonates with other results of this review about enhanced pleasure during music listening and patients’ reports of enriched emotional experiences after psilocybin treatment (Shukuroglou et al., 2023; Watts et al., 2017). The CCC circuit’s involvement might contribute to these enhanced sensory and emotional experiences, as psilocybin’s action on the claustrum through 5-HT_2A_ receptors could lead to altered states of consciousness and increased integration of sensory information, contributing to the feelings of unity and connectedness reported by patients.

The findings by Skosnik et al. (2023) suggest increased sustained neural plasticity post-psilocybin. However, with significant limitations due to the correlational nature of EEG-based measures of neural plasticity.

#### Entropic brain and relaxed beliefs under psychedelics

The demonstrated findings align with common theoretical frameworks on underlying mechanisms of psilocybin, such as the *Entropic Brain* theory and the *Relaxed Beliefs Under Psychedelics* model (Carhart-Harris, 2018; Carhart-Harris and Friston, 2019). In brief, the Entropic Brain theory proposes that the entropy (randomness) of spontaneous brain activity reflects the informational richness of conscious experiences within certain lower and upper limits (Carhart-Harris, 2018). Too little entropy can lead to rigid and inflexible functioning, while excessively high entropy might result in chaotic and unstructured states. Along with this review, recent studies indicate that psilocybin appears to elevate brain entropy by altering connectivity patterns and inducing neural plasticity (Gattuso et al., 2023). Higher entropy is hypothesized to be associated with greater sensitivity to changes and intrinsic and extrinsic influences (Carhart-Harris, 2018). It is crucial to understand that heightened entropy promotes a state of disruption and reorganization, which is vulnerable and does not per se imply a specific direction of change. Negative or hostile settings or absence of therapeutic guidance may promote dysfunctional external influences and adverse events. But, within a supportive context, heightened entropy may provide a powerful opportunity for novel and more adaptive perspectives and behaviors. This indicates that a supportive psychotherapeutic context serves a function beyond ensuring patient safety (Gründer et al., 2024b). More essentially, it may serve to utilize common factors of therapy, such as motivational clarification, resource activation, alliance bond and mastery experiences during the receptive and open state induced by psilocybin (Greenway et al., 2020). Gründer et al. (2024b) support this notion arguing that “Treatment with psychedelics is psychotherapy” (p. 231) and that its effects cannot be reduced to solely mechanistic pharmacological means.

This essential importance of context is further refined in the REBUS model. It suggests that processing of consciousness is organized hierarchically with stable, abstract beliefs at the top and basal sensory input at the bottom (Carhart-Harris and Friston, 2019). The model suggests that under the influence of psilocybin, humans may experience a relaxation of rigid top-down beliefs and increased openness for bottom-up information flow. These effects may be mediated by alterations in CTSC and CCC networks through direct pharmacological effects of psilocybin on 5-HT_2A_ receptors in these networks in complementary interaction with the wider context. The revision of pathologically overweighted beliefs is hypothesized to reinforce more flexible brain functioning and self-referential processing (Ho et al., 2020). This process can be understood through the lens of resource activation, which enhances the brain’s ability to engage existing strengths and positive experiences, thereby promoting cognitive flexibility.

Decreased connectivity within the DMN, lower brain modularity, along with reduced FC between the DMN and the NAc during music listening and DMN-amygdala during emotional face processing, as demonstrated in this review, may support the theory of relaxed top-down beliefs and less inhibitory input on bottom-up information. Simultaneous increases in FC between the amygdala and visual cortices, as well as between the DMN and visual cortices, may serve as neural correlates for increased openness to bottom-up flow of sensory information. These changes can also be interpreted as the brain clarifying and reorganizing its motivational frameworks, aligning new sensory and emotional inputs with personal goals and values—a process known as motivational clarification.

The clinical improvements associated with changes in DMN connectivity, reported in this review, for example, decreased rumination and depression severity, less perseverative cognitive errors as well as enhanced pleasure ratings during music listening align with the predictions of the REBUS model which incorporates findings concerning alterations in CTSC and CCC networks. Furthermore, REBUS’s assumptions underscore the paramount importance of therapeutic context and Gründer et al.’s (2024b) notion that psilocybin’s effects extend beyond purely pharmacological factors. Viewing these effects through the perspectives of common factors’ resource activation and motivational clarification provides a more comprehensive understanding of how psilocybin-assisted therapy can facilitate profound and lasting psychological changes.

### Emotion regulation

Psilocybin-assisted therapy induced meaningful effects on emotion regulation in the included studies correlating with clinical benefits. Overall, psilocybin reinforced participants’ ability and capacity to experience pleasant and accept unpleasant emotions. Maladaptive emotion regulation strategies such as rumination, avoidance, or suppression are clearly associated with psychopathology and depression (Aldao et al., 2010). Adaptive strategies, potentially catalyzed by psilocybin as suggested by this review, such as acceptance, reappraisal, or problem-solving are linked with better emotional and mental health.

The shift from emotional avoidance and numbness toward acceptance was among the two most mentioned mechanisms of the antidepressant effects, as reported by Watts et al. (2017) in a qualitative study of this review.

The great majority of patients in the study described their depression as a state of being cut off from their emotions and as suppressed suffering. Patients reported avoiding intense emotions due to cultural or social norms, or because they perceived their internal resources as insufficient. During the dosing session, patients were wearing eye shades and were gently encouraged to direct their attention inward and explore and feel, rather than avoid, emerging feelings and bodily sensations. The patients experienced a wide range of intense pleasant and unpleasant feelings. Some patients described the intensity of emotions as terrifying and overwhelming and that it was hard to “give in to what you’re experiencing” (Watts et al., 2017: 538).

These experiences highlight the impact of proper set and setting, as well as therapeutic guidance during the session. The therapeutic alliance bond, characterized by trust and collaboration between patient and therapist, may play a crucial role in supporting individuals to maintain an inward focus and an open state of mind during challenging experiences. The absence of such support could reduce the motivation to let go and accept difficult emotions, potentially resulting in a less pronounced antidepressant effect and increasing the risk of adverse events. This is particularly significant when traumatic memories and associated emotions are activated. The absence of these essential characteristics, that is, a therapeutic alliance that supports in confronting with difficult emotions, distinguishes recreational from therapeutic psilocybin use and illustrates why antidepressant effects observed in clinical studies cannot be transferred to other contexts.

In the qualitative study, therapeutic guidance together with psilocybin’s effects may supported patients in accepting and releasing intense emotions like deep sadness, grief, or anger (Watts et al., 2017). While this process was often described as challenging, it also led to a sense of relief afterward. This was also evident in the embodied response of patients. About half of the patients responded with intense crying during the session, with many expressing that they had not cried for a very long time, either due to emotional numbness or societal expectations. In addition, the experience of successfully navigating intense emotions can be viewed as a form of mastery by emotion regulation. Patients developed a greater sense of control and competence in managing their emotional states, contributing to long-term mental health benefits.

Although the qualitative study’s findings were preliminary and exploratory by study design, the results of the RCT-based study by Zeifman et al. (2023) replicate the trend of reduced emotional avoidance and increased acceptance following psilocybin compared to escitalopram. Notably, reductions in depression severity were significantly mediated through decreases in experiential avoidance in the psilocybin group, contrasting with no mediation effect in the escitalopram group. This effect indicates distinct antidepressant mechanisms between psilocybin and escitalopram (Zeifman et al., 2023).

Another important factor to consider when comparing psilocybin with traditional drugs for depressions arises from the qualitative study by Watts et al. (2017). Patients noted that while common drugs for depression can be helpful in crisis, they may also contribute and maintain the avoidance of underlying issues through emotional numbing (Watts et al., 2017). It could be hypothesized that common drugs for depression reduce symptoms like sadness or anxiety, but at the cost of emotional blunting. Recent research debates whether this blunting is a residual symptom of depression or an adverse effect of the medication (Christensen et al., 2022; Jawad et al., 2023; Peters et al., 2022). However, there is also research pointing out the importance of helpful social and physical environmental stimulation for positive drug effects also in classical drugs for depression (Rief et al., 2016).

This review, along with previous articles including (Wolff et al., 2020), suggests that psilocybin, when combined with proper therapeutic guidance, may intensify emotional experiences and thereby facilitate the confrontation of underlying issues. Through the lens of resource activation, motivational clarification, alliance bond experiences, and mastery by emotion regulation (Mander et al., 2013), these processes illustrate the multifaceted therapeutic potential of psilocybin-assisted therapy.

### Cognition & self-referential processing

Psilocybin induced varied changes toward a more open and resilient personality, hence less prone to depression. Furthermore, psilocybin improved mindfulness psychosocial behavior and attitudes of probands. Neural and cognitive flexibility were significantly affected by both increases and decreases in functioning, indicating a nuanced relationship between psilocybin, neural, and cognitive flexibility.

In a large meta-analysis by Kotov et al. (2010), depression has been associated with high neuroticism, introversion or low extraversion, high impulsivity, and low conscientiousness. In this review, psilocybin significantly impacted all depression-linked personality domains toward a more resilient, less psychopathology-associated personality profile (Weiss et al., 2023). The decreasing effects on Neuroticism remained stable for 6 months after receiving psilocybin, indicating lasting effects. The results replicate the findings of a prior uncontrolled open-label trial (Erritzoe et al., 2018).

Both neuroticism and hyperactivity within the DMN are associated with rigid self-referential processing and rumination (Chou et al., 2023; Zhou et al., 2020). Reductions in neuroticism could therefore be accompanied by reduced connectivity within the DMN, as suggested by previous findings of this review. This idea gains support from a recent study by Chou et al. (2023), reporting increased activity within the DMN among healthy individuals with higher neuroticism compared to those with lower scores. The direction of causality remains unclear, though. These results may grant further insight into the underlying connections between the neurological and psychological antidepressant mechanisms of psilocybin.

In addition, psilocybin significantly increased trait openness and absorption (Weiss et al., 2023). Absorption refers to an individual’s ability to get deeply immersed in sensory or mystical experiences (Witthöft et al., 2008). It includes immersion in internal mental landscapes and heightened awareness of external stimuli, such as finding meaning in nature or music (Lifshitz et al., 2019; Wild et al., 1995). High absorption is linked to paranormal beliefs and vivid spiritual and affective experiences. Notably, extreme levels of absorption have been, although weakly, linked with psychotic disorders (Glicksohn and Barrett, 2003; Lifshitz et al., 2019; Rosen et al., 2017). Findings on increased absorption align with another study in this review observing enhanced trait mindfulness, deeper meditation experience, and increases in oceanic boundlessness following psilocybin (Smigielski et al., 2019b). However, it is important to note that trait mindfulness may constitute a long-term outcome, whereas oceanic boundlessness is an acute effect of psilocybin.

Similar to the previously discussed enhanced emotional expression and experience, elevated absorption embodies a vulnerable state that may hold potent and ambiguous implications for psilocybin-assisted therapy and mental health. On one side, heightened absorption could foster therapeutic change processes by promoting intense engagement with therapeutic stimuli as well as sensory and affective experiences. On the other side, in dysfunctional environments, it may bear the risk of adverse events including psychosis. Similar considerations apply to increased trait openness. Once again, this underscores the critical importance of a supportive set, setting, and therapeutic guidance, characterized by empathy and positive regard, in realizing the potential benefits of psilocybin-induced effects. Hereby, it adds to the growing evidence and discussion suggesting that the antidepressant effect of psilocybin may not solely be attributed to the substance itself but also largely to the therapeutic context it is administered in (Gründer et al., 2024b).

Notably, within the study by Weiss et al. (2023), apart from elevated levels of absorption, there were no group differences in personality effects compared to the escitalopram control group at first sight. However, exploratory analysis revealed that pre-treatment expectancy mediated the changes in neuroticism and conscientiousness in the escitalopram group. If escitalopram expectancy was set to zero in a counterfactual model, these changes lost significance in the escitalopram condition. On the other hand, expectancy, often attributed to the effects of psilocybin therapy, did not moderate any outcomes in the psilocybin condition.

In this review, psilocybin showed mixed effects on cognitive functioning. While both divergent and convergent thinking were initially impaired, divergent thinking improved compared to baseline 1 week post-treatment (Mason et al., 2021). Furthermore, participants made fewer perseverative errors following psilocybin, indicating better cognitive flexibility (Doss et al., 2021). Notably, exploratory analysis revealed that high levels of between-network connectivity impaired cognitive functioning including convergent thinking and cognitive flexibility, while moderate levels showed a beneficial effect, suggesting a nuanced, inverted U-shaped relationship. This effectively illustrates a potential synergistic interplay between the Entropic Brain theory’s assumptions and the common factors of psychotherapy. It indicates that moderate brain entropy can help break from rigidity, inducing resource activation, motivational clarification, and mastery by self-management, whereas high entropy may lead to chaotic and less functional states.

### Connectedness

Across qualitative and quantitative studies of this review, psilocybin reinforced a sense of connectedness to the self, others, and the world (Watts et al., 2017, 2022). This change was associated with antidepressant responses and other therapeutic mechanisms, such as reduced experiential avoidance. In addition, psilocybin increased interpersonal functioning in terms of therapeutic alliance and emotional empathy compared to escitalopram and placebo.

In this review, depression has previously been described as a state of disconnection, as noted by Osler (2022). Patients’ accounts of their experiences before treatment with psilocybin align with that notion (Watts et al., 2017). They described their depression as being disconnected from their senses, self, others, and the world. In interviews 6 months after the treatment, the most common theme among patients was a process from initial disconnection toward reconnection with these qualities. An RCT-based follow-up study included in this review confirmed that psilocybin induced persistent and significant increases in connectedness scores compared to an escitalopram control group (Watts et al., 2022). While connectedness scores were increased for responders in both groups, they increased significantly more following psilocybin. The differences between groups may further indicate distinct antidepressant mechanisms of psilocybin compared to escitalopram. Moreover, the follow-up study confirmed the three-factor model of connectedness (self, others, and the world) identified in the qualitative reports, using exploratory and confirmatory factorial analyses (Watts et al., 2022).

Taking a closer look at these dimensions of connectedness helps to unravel potential antidepressant mechanisms and their interconnections. One aspect was patients’ reconnection with their *self*, namely with their sensory, bodily, and emotional experiences (Watts et al., 2017, 2022). Several patients reported engaging with their perception of self and discovering new less judgmental perspectives (Watts et al., 2017). This more accepting state of mind reportedly enabled them to reappraise old, critical self-concepts, uncovering more tender and compassionate views of themselves.

Reappraisal has been associated with potential mental health benefits (Aldao et al., 2010). Interestingly, antidepressant effects seem to depend on the success of reappraisal strategies, suggesting that attempting reappraisal creates a vulnerable state with possible outcomes in varied directions (Ford et al., 2017). This is consistent with the REBUS model and mechanisms proposed in CTSC and CCC models of psilocybin promoting a state of mind that is sensitive to positive change yet also vulnerable. This patient’s experience may help to understand the mechanism between reconnecting to self and employing adaptive emotion regulation strategies. It can be hypothesized that connectedness and less experiential avoidance facilitate mental space for the reappraisal of beliefs and emotions. Supporting this notion, Zeifman et al. (2023) found that psilocybin-induced decreases in experiential avoidance were significantly serially mediated through increases in connectedness. This effect was absent in the escitalopram control group, further highlighting the distinct therapeutic mechanism of psilocybin. It is important to consider that improvements as reported by the patients, occurred within a highly supportive context and under therapeutic guidance. Hence, the significant importance of empathy and positive regard by the therapist/setting and the possibility for emotional expression and experience also applies here.

Another domain in which patients found reconnection was in their relationship with *others*. Many patients reported a lasting sense of interconnectedness with their social groups but also strangers on the street (Watts et al., 2017). Some mentally revisited painful or traumatic relationships of their past and managed to reappraise them. They reported discovering new perspectives by feeling compassion for those who had harmed them. Many described this process as emotionally releasing, as they found new explanations for why and how these traumatic events occurred, enabling new bond experiences as well as motivational clarification.

These patients’ experiences illustrate how psilocybin may promote improvements in connectedness to others via increased motivation to confront difficult emotions and perform reappraisal strategies. It further connects to other findings of this review, suggesting improved empathy as well as therapeutic alliance and rapport following psilocybin (Murphy et al., 2022; Pokorny et al., 2017). Empathy is widely considered a key component of interpersonal functioning (Main et al., 2017). The patients’ experiences may also demonstrate how heightened empathy induced by psilocybin supported them in adopting alternative perspectives and sharing the emotional state of others, thereby gaining a better understanding of their motivations.

Psilocybin’s potential to enhance private and therapeutic relationships, as indicated by the study of Murphy et al. (2022), could significantly advance our understanding of its mechanisms and inform its application in therapy. The therapeutic relationship is universally recognized as the most fundamental factor and mediator of change in psychotherapy (Baier et al., 2020; Norcross and Lambert, 2018). A safe and stable therapeutic relationship also appears to be an essential element in psilocybin-assisted therapy, given the heightened openness and potential vulnerability demonstrated in this review. Moreover, lasting psilocybin-induced improvements in therapeutic alliance may foster positive change in regular psychotherapy. Future studies investigating the long-term effects of psilocybin on therapeutic alliance are needed to validate this hypothesis, though.

Lastly, the studies reported a reconnection with the *world.* As conceptualized by Watts et al. (2022), this includes various qualities such as humanity, universal love, or nature. It can be hypothesized that experiencing profound feelings of connectedness with the world counteracts rigid disconnected states of depression. One patient said, “I felt like sunshine twinkling through leaves, I *was* nature” (Watts et al., 2017). Deep immersion into sensory experiences of nature, as described by this patient, resonate with the findings on increased trait absorption by Weiss et al. (2023), earlier described in this review. Moreover, nature connectedness was found to promote adaptive emotion regulation strategies and reduce stress levels further underscoring its importance and legitimacy in novel treatment approaches for depression (Bakir-Demir et al., 2021).

### Integration and future outlook

**Figure 2.**
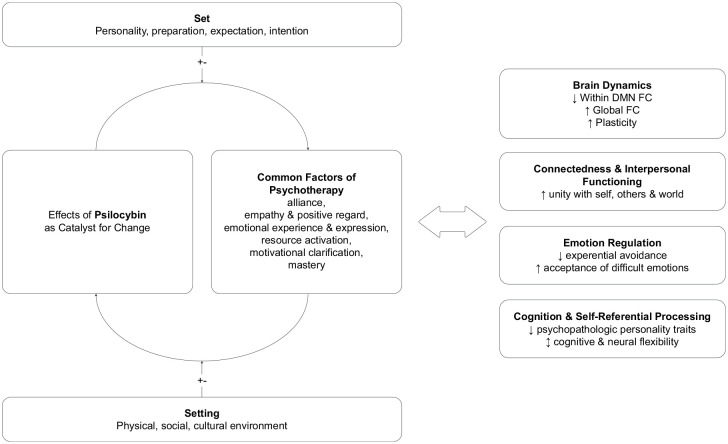
Conceptualization of review findings and candidates for antidepressant mechanisms. *Note.* Aspects of set and setting according to Hartogsohn (2017). Common factors of psychotherapy according to Norcross and Lambert (2018) and Wucherpfennig et al. (2024). The key outcomes on the right correspond to the primary domains identified in this review. ↑ = increasing effect; ↓ = decreasing effect; ↕ = mixed or dose-dependent effect.

Figure 2 integrates the findings of this systematic review and proposes potential candidates for mechanisms underlying the antidepressant effect of psilocybin-assisted treatment approaches.

The results suggest an interactive conglomerate of psilocybin, set & setting, and common factors of psychotherapy contributing to the observed effects. The primary outcomes on the right were linked to antidepressant responses through correlations and serial mediation effects reported in this review, suggesting them as potential mechanisms of change within psilocybin-assisted psychotherapy. However, it is important to note that, at this stage of research, no directional causality can be claimed, and it is likely that unknown additional factors have been overlooked by recent studies.

Hence, the findings appear to expand upon and complement the notion proposed by Goodwin et al. (2024), who propose that psilocybin predominantly exerts a mechanistic pharmacological effect and does not necessarily require psychotherapy to achieve antidepressant outcomes. Consequently, this review suggests a multileveled interplay between the direct pharmacological effects of psilocybin, acting as a catalyst for common factors of psychotherapy as suggested by Gründer et al. (2024b), and environmental variables such as set and setting. The findings highlight the need for an integrative approach that reflects multiple perspectives on psilocybin’s antidepressant mechanisms, as illustrated in Figure 2. Future studies are encouraged to expand upon this concept with additional data as more qualified trials become available and are invited to embrace multiple perspectives (e.g., pharmacological, molecular, epigenetic, psychological, and systemic) rather than focusing solely on reductionist approaches.

In addition, changes in self-referential processing, emotion regulation, connectedness, and interpersonal functioning can be viewed through the lens of personality functioning as defined by criterion A of the alternative model for personality disorders in the DSM-5 which includes self-functioning (identity and self-direction) and interpersonal functioning (empathy and intimacy) (Sharp and Wall, 2021). The review’s findings indicate that psilocybin appears to enhance these aspects by promoting changes in self-referential processing, personality, empathy, and intimate connections with oneself, others, and the world (Pokorny et al., 2017; Smigielski et al., 2019b; Watts et al., 2017, 2022; Weiss et al., 2023).

Recent investigations could show that problems in aspects of personality functioning such as identity, emotion regulation, and internal models of relationships are central to a wide range of psychopathology (Kerber et al., 2024).

In addition to the existing connections between change processes in psilocybin-assisted therapy and frameworks such as the Research Domain Criteria (Kelly et al., 2021; Pouyan et al., 2022), future research should investigate changes in personality functioning in relation to alterations of brain dynamics, self-referential processing, emotion regulation, connectedness, and interpersonal functioning.

### Limitations and strengths

This systematic review has various strengths and limitations. To begin with the strengths, it offers an overview of psilocybin’s potential antidepressant mechanisms across diverse domains, contributing to a holistic understanding of its effects and underlying interconnections. Moreover, integrating the findings of this review into existing frameworks beyond psilocybin-related research, such as common factors of psychotherapy and transdiagnostic factors for psychopathology such as personality functioning helps connect new findings with established knowledge. However, as this field of research just emerges after decades of stagnation, the quality of studies is very heterogeneous. While 11 studies were based on RCTs or NRCTs, 5 studies relied on single-arm open-label trials, due to the lack of alternatives. The results stemming from uncontrolled studies must therefore be cautiously interpreted as preliminary. Despite the explorative character, the results may still inform hypotheses for future confirming research. The broad range of mechanisms examined may unveil interconnections but also pose comparability challenges. Several included studies were novel or unique in their field, lacking comparable counterparts. The four domains that this review introduces for clarity are not meant to be exhaustive and need to be validated with factorial analysis.

Also, it is fundamental to declare that, at this stage of the research, claims to causality are minimal or non-existent. The mechanisms of change presented in this review should be viewed as potential preliminary candidates. While direct correlations and mediation analysis with the antidepressant effects provide hints of possible causality, the reported effects across all four domains may well be byproducts of the actual, possibly unknown or overlooked, mechanisms.

The nature of psilocybin’s effects demonstrated in this review highlights the importance of a supportive therapeutic context. However, many studies insufficiently report how they employed set, setting, and therapeutic guidance. Future studies should put more emphasis on the details of the therapeutic concept of preparation, dosing, and integration sessions, given its essential role.

A central methodological limitation among all included studies are potential problems with blinding and pre-treatment expectancy. These have been widely discussed as a source of potential bias for novel treatment approaches including psilocybin (Gründer et al., 2024a). Although RCTs are double-blinded per design, the strong effects of psilocybin often break the blinding to practitioners and participants creating various sources of bias, including both placebo and nocebo effects (Mertens et al., 2022). The disappointment in expectancy within control groups may negatively influence treatment outcomes and create false effects. However, a recent study found that despite higher initial expectancy for psilocybin, its association with treatment outcomes was only significant in the escitalopram control group (Szigeti et al., 2024).

Moving forward, studies should consistently address these challenges by controlling for pre-treatment expectancy like Weiss et al. (2023) or offering a potentially effective dose to control group participants post-primary study endpoints to reduce nocebo effects, as proposed by Mertens et al. (2022).

## Conclusion

In summary, this review suggests that psilocybin acts as a potent catalyst for changes across various domains, including brain dynamics, emotion regulation, self-referential processing, and interpersonal functioning. These effects proved to be interconnected and associated with clinical improvements. Evidence suggests that psilocybin promotes a state of consciousness characterized by heightened openness, flexibility, and greater ability and acceptance of emotional experiences. Moreover, a renewed sense of connectedness to the self, others, and the world emerged as a key experience of treatment with psilocybin. Consistent reports indicate significant alterations in underlying brain dynamics, marked by reduced global and DMN modularity and increasing connectivity between networks. The findings align with the assumptions of the Entropic Brain theory as well as REBUS, CTSC, and CCC models.

Collectively, these effects indicate parallels to adaptive emotion regulation strategies and common factors of effectiveness in psychotherapy, such as alliance bond experiences, perceived empathy, positive regard from the therapist or setting, opportunities for emotional expression and experience, activation of resources, motivational clarification, and mastery through self-management and emotion regulation.

Together, these changes may create a fertile yet vulnerable window for change processes, strongly emphasizing the essential importance of supportive set, setting and therapeutic guidance in fostering the benefits of psilocybin. Consequently, the results suggest that psilocybin, within a supportive context, may induce antidepressant effects by leveraging the interplay between neurobiological mechanisms and common psychotherapeutic factors. These findings complement the view of purely pharmacological effects, supporting a multileveled approach that reflects various relevant dimensions of therapeutic change, including neurobiological, psychological, and environmental factors.

## Supplemental Material

sj-docx-1-jop-10.1177_02698811241312866 – Supplemental material for Catalyst for change: Psilocybin’s antidepressant mechanisms—A systematic review Supplemental material, sj-docx-1-jop-10.1177_02698811241312866 for Catalyst for change: Psilocybin’s antidepressant mechanisms—A systematic review by Joshua Liebnau, Felix Betzler and André Kerber in Journal of Psychopharmacology
