# Supplementary material for: Catalyst for change: Psilocybin’s antidepressant mechanisms—A systematic review
Source: J Psychopharmacol. 2025 Jan 20;39(5):397–415. doi: 10.1177/02698811241312866 (PMC12099018; doi:10.1177/02698811241312866)
Supplement: sj-docx-1-jop-10.1177_02698811241312866 – Supplemental material for Catalyst for change: Psilocybin’s antidepressant mechanisms—A systematic review [file sj-docx-1-jop-10.1177_02698811241312866.docx]

# **Supplementary Material**

## Search Terms

psilocybin AND

connectedness

OR "neural plasticity"

OR "functional connectivity"

OR openness

OR "psychological flexibility"

OR flexibility

OR avoidance

OR alliance

OR collaboration

OR “goal consensus”

OR cohesion

OR empathy

OR “positive regard”

OR “positive affirmation”

OR "experiential avoidance"

OR "motivational clarification"

OR "problem activation"

OR "resource activation"

OR mastery

OR "emotional activation"

#

## Source Trials of Included Studies

## Table 1.

*Source Trials of Included Studies of the Systematic Review.*

| Trial ID | Clinical Trial | Included Studies |
| --- | --- | --- |
| 1 | Carhart-Harris et al. (2017) | Carhart-Harris et al. (2017), Daws et al. (2022), Mertens et al. (2020), Shukuroglou et al. (2023), Watts et al. (2017) |
| 2 | Carhart-Harris et al. (2021) | Murphy et al. (2022), Watts et al. (2022), Weiss et al. (2023), Zeifman et al. (2023) |
| 3 | Skosnik et al. (2023) | Skosnik et al. (2023) |
| 4 | Smigielski et al. (2019b) | Smigielski et al. (2019a), Smigielski et al. (2019b) |
| 5 | Davis et al. (2021) | Doss et al. (2021) |
| 6 | Mason et al. (2020) | Mason et al. (2021) |
| 7 | Pokorny et al. (2017) | Pokorny et al. (2017) |

*Note.* All included studies of the systematic review along with their source trials and trial IDs.

## Risk of Bias Assessment Table 2.

*Risk of Bias Assessment of the Included RCTs.*

| Trial | Reference | Risk of Bias Sources | | | | | |  | Overall Risk of Bias Judgment |
| --- | --- | --- | --- | --- | --- | --- | --- | --- | --- |
|  |  | Randomization Process | Deviations From Intended Intervention | Missing Outcome Data | Measurement of the Outcome | Selection of the Reported Result | Other Sources of Bias |  |  |
| *Brain Dynamics* | | | | | | | | | |
| 1, 2 | Daws et al., 2022 | Low | Some concerns | Low | Low | Low | Low |  | Some concerns |
| 4 | Smigielski et al., 2019a | Low | Some concerns | Low | Some concerns | Low | Low |  | Some concerns |
| *Emotion Regulation* | | | | | | |  |  |  |
| 2 | Zeifmann et al., 2023 | Low | Some concerns | Low | Some concerns | Low | Low |  | Some concerns |
| *Cognition & Self-Referential Processing* | | | | | | |  |  |  |
| 2 | Weiss et al., 2023 | Low | Low | Some concerns | Some concerns | Low | Low |  | Some concerns |
| 4 | Smigielski et al., 2019b | Low | Some concerns | Low | Low | Some concerns | Low |  | Some concerns |
| 5 | Doss et al., 2021 | Low | Some concerns | Low | Low | Some concerns | Low |  | Some concerns |
| 6 | Mason et al., 2021 | Low | Some concerns | Low | Low | Low | Low |  | Some concerns |
| *Connectedness & Interpersonal Functioning* | | | | | | |  |  |  |
| 2 | Watts et al., 2022 | Low | Some concerns | Low | Low | Low | Low |  | Some concerns |
| 2 | Murphy et al., 2022 | Low | Some concerns | Low | Low | Low | Low |  | Some concerns |
| 7 | Pokorny et al., 2017 | Low | Some concerns | Some concerns | Some concerns | Low | Low |  | Some concerns |

*Note.* Risk of bias assessment using the Revised Cochrane risk-of-bias tool for randomized trials (RoB 2).

**Table 3.**

*Risk of Bias Assessment of the Included NRCTs.*

| Trial | Reference | Risk of Bias Sources | | | | | | |  | Overall Risk of Bias Judgment |
| --- | --- | --- | --- | --- | --- | --- | --- | --- | --- | --- |
|  |  | Confounding | Selection of Participants Into the Study | Classification of Interventions | Deviations From the Intended intervention | Missing Data | Measurement of Outcomes | Selection of the Reported Result |  |  |
| *Brain Dynamics* | | | | | | | | | |  |
| 1 | Carhart-Harris et al., 2017 | Moderate | Low | Low | Low | Moderate | Low | Moderate |  | Moderate |
| 1, 2 | Daws et al., 2022 | Moderate | Low | Low | Low | Moderate | Low | Moderate |  | Moderate |
| 3 | Skosnik et al., 2023 | Moderate | Low | Low | Low | Low | Moderate | Low |  | Moderate |
| *Emotion Regulation* | | | | | | | | | |  |
| 1 | Mertens et al., 2020 | Moderate | Low | Low | Low | Moderate | Moderate | Moderate |  | Moderate |
| 1 | Shukuroglou et al., 2023 | Moderate | Low | Low | Low | Moderate | Moderate | Moderate |  | Moderate |

*Note.* Risk of bias assessment using The Risk Of Bias In Non-randomized Studies – of Interventions (ROBINS-I) tool.

**Table 4.**

*Risk of Bias Assessment of the Included Qualitative Study.*

| Reference | Trial | CASP Checklist Questions | Assessment |
| --- | --- | --- | --- |
| *Connectedness & Interpersonal Functioning* | | | |
| Watts et al., 2017 | 1 | Was there a clear statement of the aims of the research? | Yes |
|  |  | Is a qualitative methodology appropriate? | Yes |
|  |  | Was the research design appropriate to address the aims of the research? | Yes |
|  |  | Was the recruitment strategy appropriate to the aims of the research? | Yes |
|  |  | Was the data collected in a way that addressed the research issue? | Yes |
|  |  | Has the relationship between researcher and participants been adequately considered? | Yes |
|  |  | Have ethical issues been taken into consideration? | Yes |
|  |  | Was the data analysis sufficiently rigorous? | Can’t tell |
|  |  | Is there a clear statement of findings? | Yes |
|  |  | How valuable is the research? | Significantly valuable, as it identified several potential future fields of quantitative research not previously recognized. It inspired several later studies e.g., Watts et al., 2022 or Zeifmann et al., 2023. |

*Note.* Risk of bias assessment using the Critical Appraisal Skills Programme

**References**

Carhart-Harris R, Giribaldi B, Watts R, et al. (2021) Trial of Psilocybin versus Escitalopram for Depression. *New England Journal of Medicine* 384(15): 1402–1411.

Carhart-Harris RL, Roseman L, Bolstridge M, et al. (2017) Psilocybin for treatment-resistant depression: fMRI-measured brain mechanisms. *Scientific reports* 7(1). England: 13187.

Davis AK, Barrett FS, May DG, et al. (2021) Effects of Psilocybin-Assisted Therapy on Major Depressive Disorder: A Randomized Clinical Trial. *JAMA Psychiatry* 78(5): 481.

Daws RE, Timmermann C, Giribaldi B, et al. (2022) Increased global integration in the brain after psilocybin therapy for depression. *Nature medicine* 28(4). United States: 844–851.

Doss M, Povazan M, Rosenberg M, et al. (2021) Psilocybin therapy increases cognitive and neural flexibility in patients with major depressive disorder. *TRANSLATIONAL PSYCHIATRY* 11(1).

Mason NL, Kuypers KPC, Müller F, et al. (2020) Me, myself, bye: regional alterations in glutamate and the experience of ego dissolution with psilocybin. *Neuropsychopharmacology* 45(12): 2003–2011.

Mason NL, Kuypers KPC, Reckweg JT, et al. (2021) Spontaneous and deliberate creative cognition during and after psilocybin exposure. *Translational psychiatry* 11(1). United States: 209.

Mertens LJ, Wall MB, Roseman L, et al. (2020) Therapeutic mechanisms of psilocybin: Changes in amygdala and prefrontal functional connectivity during emotional processing after psilocybin for treatment-resistant depression. *Journal of Psychopharmacology* 34(2). Sage Publications: 167–180.

Murphy R, Kettner H, Zeifman R, et al. (2022) Therapeutic Alliance and Rapport Modulate Responses to Psilocybin Assisted Therapy for Depression. *FRONTIERS IN PHARMACOLOGY* 12.

Pokorny T, Preller KH, Kometer M, et al. (2017) Effect of Psilocybin on Empathy and Moral Decision-Making. *The international journal of neuropsychopharmacology* 20(9). England: 747–757.

Shukuroglou M, Roseman L, Wall M, et al. (2023) Changes in music-evoked emotion and ventral striatal functional connectivity after psilocybin therapy for depression. *Journal of Psychopharmacology* 37(1). Sage Publications: 70–79.

Skosnik PD, Sloshower J, Safi-Aghdam H, et al. (2023) Sub-acute effects of psilocybin on EEG correlates of neural plasticity in major depression: Relationship to symptoms. *Journal of Psychopharmacology* 37(7). Sage Publications: 687–697.

Smigielski L, Kometer M, Scheidegger M, et al. (2019) Characterization and prediction of acute and sustained response to psychedelic psilocybin in a mindfulness group retreat. *Scientific reports* 9(1). England: 14914.

Smigielski L, Scheidegger M, Kometer M, et al. (2019) Psilocybin-assisted mindfulness training modulates self-consciousness and brain default mode network connectivity with lasting effects. *NeuroImage* 196. Elsevier Science: 207–215.

Watts R, Day C, Krzanowski J, et al. (2017) Patients’ Accounts of Increased “Connectedness” and “Acceptance” After Psilocybin for Treatment-Resistant Depression. *Journal of Humanistic Psychology* 57(5): 520–564.

Watts R, Kettner H, Geerts D, et al. (2022) The Watts Connectedness Scale: A new scale for measuring a sense of connectedness to self, others, and world. *Psychopharmacology* 239(11). Springer: 3461–3483.

Weiss B, Ginige I, Shannon L, et al. (2023) Personality change in a trial of psilocybin therapy v. escitalopram treatment for depression. *PSYCHOLOGICAL MEDICINE*. Epub ahead of print 2 June 2023. DOI: 10.1017/S0033291723001514.

Zeifman RJ, Wagner AC, Monson CM, et al. (2023) How does psilocybin therapy work? An exploration of experiential avoidance as a putative mechanism of change. *Journal of Affective Disorders* 334. Elsevier Science: 100–112.
